# Effect of Tourmaline Addition on the Anti-Poisoning Performance of MnCeO_x_@TiO_2_ Catalyst for Low-Temperature Selective Catalytic Reduction of NO*_x_*

**DOI:** 10.3390/molecules29174079

**Published:** 2024-08-28

**Authors:** Zhenzhen Zhao, Liyin Wang, Xiangqing Lin, Gang Xue, Hui Hu, Haibin Ma, Ziyu Wang, Xiaofang Su, Yanan Gao

**Affiliations:** 1 School of Advanced Agricultural Science, Weifang University, Weifang 261061, China; 20230012@wfu.edu.cn (Z.Z.); 18769313866@163.com (X.L.); 18861906532@163.com (Z.W.); 2 Institute of Power Source and Ecomaterials Science, Hebei University of Technology, Tianjin 300130, China; 15122281505@163.com; 3 Key Laboratory of Ministry of Education for Advanced Materials in Tropical Island Resources, Hainan University, Haikou 570228, China; hhu@hainanu.edu.cn (H.H.); sxf@hainanu.edu.cn (X.S.); 4 School of Chemistry, Chemical & Environmental Engineering, Weifang University, Weifang 261061, China

**Keywords:** nitrogen oxides, denitration catalyst, tourmaline, anti-poisoning performance, flue gas

## Abstract

In view of the flue gas characteristics of cement kilns in China, the development of low-temperature denitrification catalysts with excellent anti-poisoning performance has important theoretical and practical significance. In this work, a series of MnCeO*_x_*@TiO_2_ and tourmaline-containing MnCeO*_x_*@TiO_2_-T catalysts was prepared using a chemical pre-deposition method. It was found that the MnCeO*_x_*@TiO_2_-T2 catalyst (containing 2% tourmaline) exhibited the best low-temperature NH_3_-selective catalytic reduction (NH_3_-SCR) performance, yielding 100% NO*_x_* conversion at 110 °C and above. When 100–300 ppm SO_2_ and 10 vol.% H_2_O were introduced to the reaction, the NO*_x_* conversion of the MnCeO*_x_*@TiO_2_-T2 catalyst was still higher than 90% at 170 °C, indicating good anti-poisoning performance. The addition of appropriate amounts of tourmaline can not only preferably expose the active {001} facets of TiO_2_ but also introduce the acidic SiO_2_ and Al_2_O_3_ components and increase the content of Mn^4+^ and O_α_ on the surface of the catalyst, all of which contribute to the enhancement of reaction activity of NH_3_-SCR and anti-poisoning performance. However, excess amounts of tourmaline led to the formation of dense surface of catalysts that suppressed the exposure of catalytic active sites, giving rise to the decrease in catalytic activity and anti-poisoning capability. Through an in situ DRIFTS study, it was found that the addition of appropriate amounts of tourmaline increased the number of Brønsted acid sites on the catalyst surface, which suppressed the adsorption of SO_2_ and thus inhibited the deposition of NH_4_HSO_4_ and (NH_4_)_2_HSO_4_ on the surface of the catalyst, thereby improving the NH_3_-SCR performance and anti-poisoning ability of the catalyst.

## 1. Introduction

With the development of human society, the composition of the atmosphere is being changed, which has led to serious environmental problems [1,2,3]. Nitrogen oxides are one of the main pollutants in the prevention and control of atmospheric environmental pollution. The emission of NO*_x_* in the cement industry has become the third-largest source of pollution after thermal power plants and motor vehicle exhaust. Therefore, there is an urgent need to research and develop efficient strategies to reduce NO*_x_* emissions. Compared to the power industry, the flue gas of cement kilns in China is characterized by high oxygen content, high humidity, and low temperature window (80–200 °C). Ammonia-selective catalytic reduction of NO*_x_* (NH_3_-SCR) is considered to be an effective strategy for reducing NO*_x_* emissions [4]. Composite metal oxide catalysts such as manganese-based and cerium-based oxides exhibit good denitrification capability in the low-temperature section, but their anti-poisoning (mainly refereeing to SO_2_ and water vapor) performance is found to be poor [5,6,7]. The deposition of (NH_4_)_2_SO_4_ and NH_4_HSO_4_ on the surface of the catalyst blocks the exposure of active sites and water vapor can lead to partially destructing acid sites to decrease the number of available active sites. Therefore, research and exploitation of NH_3_-SCR catalysts with high activity and selectivity, and good anti-poisoning capability as well as broad range of operating temperature have become the focus of current research in the field.

To date, various strategies have been attempted to enhance the catalytic activity and the anti-poisoning performance of NH_3_-SCR catalysts. Metal oxides, such as V_2_O_5_, Fe_3_O_4_, and MnO*_x_* and bimetallic compounds, such as MnO*_x_*/TiO_2_ [8], CrO*_x_*/TiO_2_ [9], and CuO/MnO*_x_* [10] Zr/Ce phosphates [11], have been found to be highly active and selective catalysts in NH_3_-SCR. For instance, Tang et al. reported that nearly 100% N_2_ selectivity was obtained on MnO*_x_*-CeO_2_ catalyst at 100–150 °C [12], but the selectivity was relatively low on MnO*_x_* in the same temperature range [13,14]. It has been reported that the strong interaction between CeO_2_ and SO_2_ promoted the formation of CeSO_4_ or Ce_2_(SO_4_)_3_. Thus, using CeO_2_ as a sacrificial site is effective to reduce the sulfation of the main active phase [15]. Han et al. [16] found that the mesoporous structure and the presence of metal oxides in the catalyst can greatly promote the decomposition of ammonium sulfate deposited on the surface of the catalyst during the SCR reaction. Yu et al. [17] found that the mesoporous TiO_2_ shell of the TiO_2_@Fe_2_O_3_ monolithic catalyst promoted the decomposition of NH_4_HSO_4_. Han et al. [18] successfully coated mesoporous TiO_2_ layers on carbon-nanotube-supported MnO*_x_* and CeO*_x_* nanoparticles to obtain a core–shell catalyst. The mesoporous TiO_2_ sheaths enhanced the acid strength and quantity, thus leading to higher activity and a wider operating temperature window. Additionally, the strong interaction between MnO*_x_*, CeO*_x_* and the mesoporous TiO_2_ can inhibit the aggregation of MnO*_x_* and CeO*_x_* nanoparticles, affording the catalyst with good stability.

Tourmaline shows several unique properties, including far-infrared radiation, spontaneous polarity, and the release of negative ions. Thanks to these properties, tourmaline has been widely used as a function material in the field of catalysis [19,20,21,22]. For instance, Hu et al. [23] found that the presence of electric field and far-infrared radiation of tourmaline can promote the synthesis of Pd nanoparticles with smaller size, weaken the Pd-O bond, and increase molecular vibration and migration rate, thus improving the electrocatalytic performance of Pd for formic acid electrooxidation. Luo et al. [24] used a co-precipitation method to prepare MnO*_x_*/TiO_2_ catalysts with addition of tourmaline. When the tourmaline content was 10%, the conversion of NO*_x_* at 200 °C exceeds 97%, which was about 7% higher than that of the undoped counterpart. Due to the permanent electrode of tourmaline, it was reported that tourmaline can effectively promote the dispersion of MnO*_x_*/TiO_2_ catalyst, increasing the number of acidic sites and changed the valence distribution of manganese ions in the product, thereby accelerating the diffusion of manganese ions and leading to accelerated redox reactions. The far-infrared radiation and spontaneous polarity of tourmaline can generate electric fields, which tends to weaken or destroy the hydrogen bonds of water. During the reaction, many hydrogen bonds will be broken and rearranged. Therefore, the water clusters will become smaller, reducing the clustering effect of water molecules caused by hydrogen bonds [25]. Despite these achievements, it remains a great challenge to develop low-temperature SCR catalysts with high catalytic activity SO_2_/H_2_O tolerance and stability to eliminate NO*_x_*.

Based on the above, we herein prepared a MnO*_x_*/CeO_2_ bimetallic oxide catalyst and dopped mesoporous TiO_2_ on the surface of the MnO*_x_*/CeO_2_ with the addition of different amounts of tourmaline. The NO*_x_* conversion and anti-poisoning performance of the obtained MnCeO*_x_*@TiO_2_ and MnCeO*_x_*@TiO_2_-T were examined. Among all catalysts, MnCeO*_x_*@TiO_2_-T2 exhibited the best catalytic performance. It was found that the addition of appropriate amounts of tourmaline enhanced the catalytic activity and increased the number of Brønsted acid sites on the catalyst surface, and inhibited the deposition of ammonium sulfate, thus improving the SCR reaction and anti-poisoning ability of the catalyst.

## 2. Results and Discussion

### 2.1. NO_x_ Reduction and SO_2_ Tolerance

**Figure 1:**
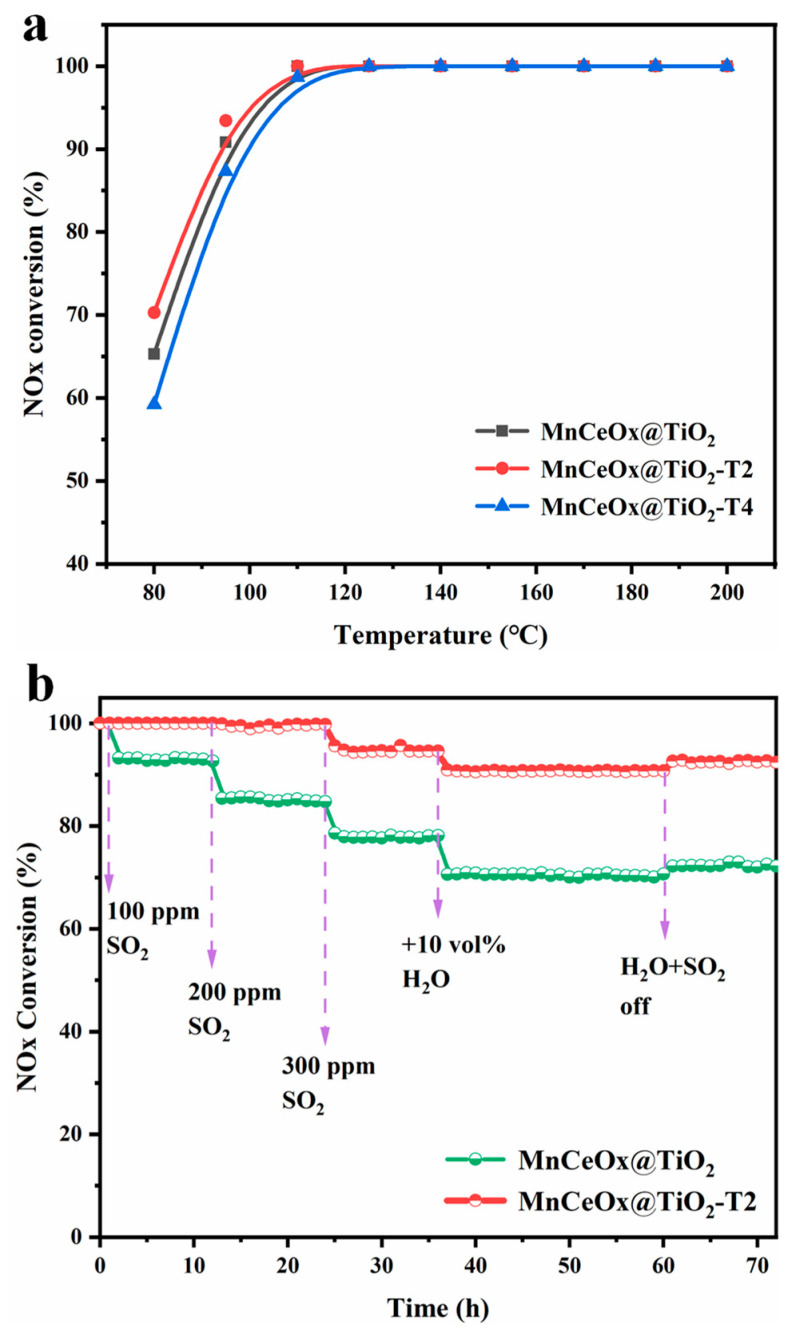
(**a**) Reversion of NO*_x_* catalyzed by MnCeO*_x_*@TiO_2_ with different amounts of tourmaline. (**b**) Anti-poisoning performance of MnCeO*_x_*@TiO_2_ and MnCeO*_x_*@TiO_2_-T2 catalysts investigated at 170 °C.

Figure 1a shows the effect of tourmaline addition on the denitration performance of MnCeO*_x_*@TiO_2_ catalyst. It can be seen that after adding tourmaline, the MnCeO*_x_*@TiO_2_-T2 catalyst exhibited the best denitration performance compared to other catalysts at a temperature ranging from 80 to 110 °C. The MnCeO*_x_*@TiO_2_-T2 catalyst showed a 75% NO*_x_* conversion at 80 °C, which is higher than that of the MnCeO*x*@TiO_2_ catalyst (65%) at the same temperature. A 100% NO*_x_* conversion was achieved by MnCeO*_x_*@TiO_2_-T1 and MnCeO*_x_*@TiO_2_-T2 in the temperature range of 110~200 °C. However, when the tourmaline content is 3% and 4%, the NO*_x_* conversion of MnCeO*_x_*@TiO_2_-T3 and MnCeO*_x_*@TiO_2_-T4 is lower than that of MnCeO*_x_*@TiO_2_-T2 at temperature less than 110 °C, indicating that the amount of added tourmaline affected the denitration activity of the catalyst.

Considering that MnCeO*_x_*@TiO_2_-T2 exhibited the best catalytic activity, the catalyst was mainly investigated in the following research. The anti-poisoning performance of MnCeO*_x_*@TiO_2_ and MnCeO*_x_*@TiO_2_-T2 was examined at 170 °C, and the results are shown in Figure 1b. For MnCeO*_x_*@TiO_2_, when 100 ppm SO_2_ was introduced, the NO*_x_* conversion decreased from 100% to 93% and then remained stable for 12 hours. As the SO_2_ concentration increased to 200 ppm, the NO*_x_* conversion decreased to 85% and remained stable for 12 h. As the SO_2_ concentration further increased to 300 ppm, the NO*_x_* conversion dropped to 78%, and remained stable for another 12 h. After 10 vol.% H_2_O was introduced, the NO*_x_* conversion further dropped to 70%. In contrast, after 100–200 ppm of SO_2_ was introduced into the feed, the NO*_x_* conversion of MnCeO*_x_*@TiO_2_-T2 catalyst was still 100% and remained unchanged and stable for 24 h. As the SO_2_ concentration increased to 300 ppm, the NO*_x_* conversion of MnCeO*_x_*@TiO_2_-T2 decreased slightly from 100% to 95%. After 10 vol.% H_2_O was further introduced, the NO*_x_* conversion decreased to about 90%. The decrease in NO*_x_* conversion can be attributed to the sulfation of metal oxides and the deposition of ammonium sulfate. After cutting off H_2_O and SO_2_, the NO*_x_* conversion of MnCeO*_x_*@TiO_2_ and MnCeO*_x_*@TiO_2_-T2 rebounded to 72% and 93%, respectively, and remained stable for 12 h. These results show that the presence of sulfur species on the surface of catalysts seriously affected their catalyst performance, and the poisoning of the catalysts was substantially irreversible. Moreover, it can also be seen that the MnCeO*_x_*@TiO_2_-T2 catalyst exhibited better anti-poisoning performance than the MnCeO*_x_*@TiO_2_ catalyst. A comparison with the previous reports was listed in Table S1.

### 2.2. Research on Crystal Structure and Morphology

**Figure 2:**
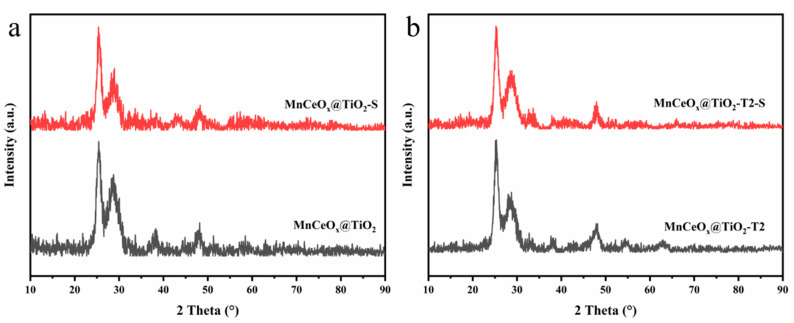
PXRD patterns of (**a**) MnCeO*x*@TiO_2_ and (**b**) MnCeO*x*@TiO_2_-T2 catalysts before and after SO_2_ poisoning.

The crystal structures of MnCeO*_x_*@TiO_2_ and MnCeO*_x_*@TiO_2_-T2 catalysts before and after poisoning are shown in Figure 2. It can be seen that both catalysts exhibited characteristic peaks of anatase TiO_2_ (PDF#21-1272) [26] and CeO_2_ (PDF#43-1002) [27]. The peaks at 25.3, 37.8, and 48.1° are due to the (101), (004), and (200) facets of TiO_2_, respectively, and the peak at 28.3° can be attributed to the (111) facet of CeO_2_. No characteristic peaks of MnO*_x_* were observed in either powder X-ray diffraction (PXRD) pattern, suggesting that amorphous MnO*_x_* was highly dispersed within the catalysts, which is favorable for improving their catalytic performance [28,29]. After the addition of 2% tourmaline, the intensity ratio of I(004)/I(200) of TiO_2_ in MnCeO*_x_*@TiO_2_-T2 pattern was slower than that in MnCeO*_x_*@TiO_2_ because MnCeO*_x_*@TiO_2_-T2 demonstrated a decrease in the (004) diffraction intensity and an increasement in the (200) intensity compared with that of MnCeO*_x_*@TiO_2_, which showed that MnCeO*_x_*@TiO_2_-T2 possesses more exposed {001} facets [30], which can increase the concentration of surface adsorbed oxygen of the catalyst and increase the surface acidity, giving rise to better NO*_x_* conversion [31]. After the SO_2_ poisoning, the CeO_2_ diffraction peaks of MnCeO*_x_*@TiO_2_ catalyst were weakened. The decreased diffraction peak intensity of CeO_2_ could be due to the formation of CeSO_4_. As mentioned above, the CeO_2_ acts as a sacrificial site that can reduce the sulfation of the main active phase [15]. In contrast, the diffraction peaks of MnCeO*_x_*@TiO_2_-T2 catalyst were not obviously changed, suggesting that the addition of an appropriate amount of tourmaline can suppress the adsorption of SO_2_, thereby demonstrating the outstanding sulfur resistance of the catalyst [32].

**Figure 3:**
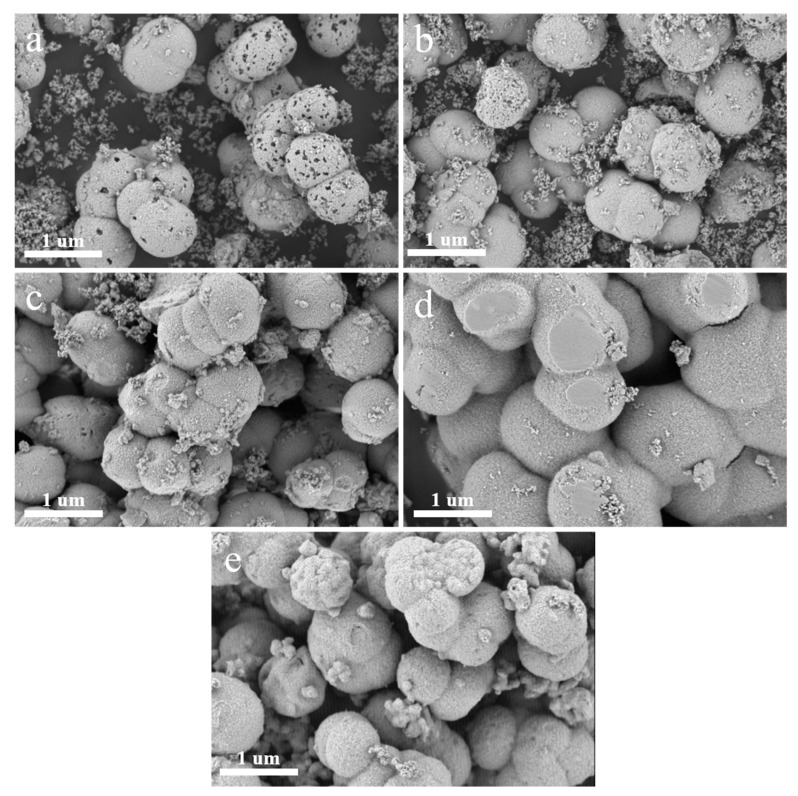
SEM images of MnCeO*_x_*@TiO_2_ catalysts with different amounts of tourmaline (**a**) MnCeO*_x_*@TiO_2_; (**b**) MnCeO*_x_*@TiO_2_-T1; (**c**) MnCeO*_x_*@TiO_2_-T2; (**d**) MnCeO*_x_*@TiO_2_-T3; and (**e**) MnCeO*_x_*@TiO_2_-T4.

The scanning electron microscopy (SEM) images of MnCeO*_x_*@TiO_2_ with different amounts of tourmaline are shown in Figure 3. It can be seen that as the added amount of tourmaline increases, the pores on the surface of spherical catalysts disappear gradually and the MnCeO*_x_*@TiO_2_ particles become more and more dense, which is consistent with the PXRD results that showed enhanced crystallinity of both TiO_2_ and CeO_2_ (Figure S1). This is actually unfavorable for the NH_3_-SCR. Since the spontaneous polarization characteristics of tourmaline would change the crystal growth during the catalyst preparation process, the morphology of the catalyst was changed with the addition of different amounts of tourmaline, which further affects the denitration performance of the catalyst. The catalytic experiments showed that when 3% or 4% tourmaline was added, the catalytic performance of MnCeO*_x_*@TiO_2_-T3 and MnCeO*_x_*@TiO_2_-T4 was decreased, which may be attributed to the dense surface of the catalyst that blocks the exposure of active sites and suppresses the mass transfer and diffusion of substance.

**Figure 4:**
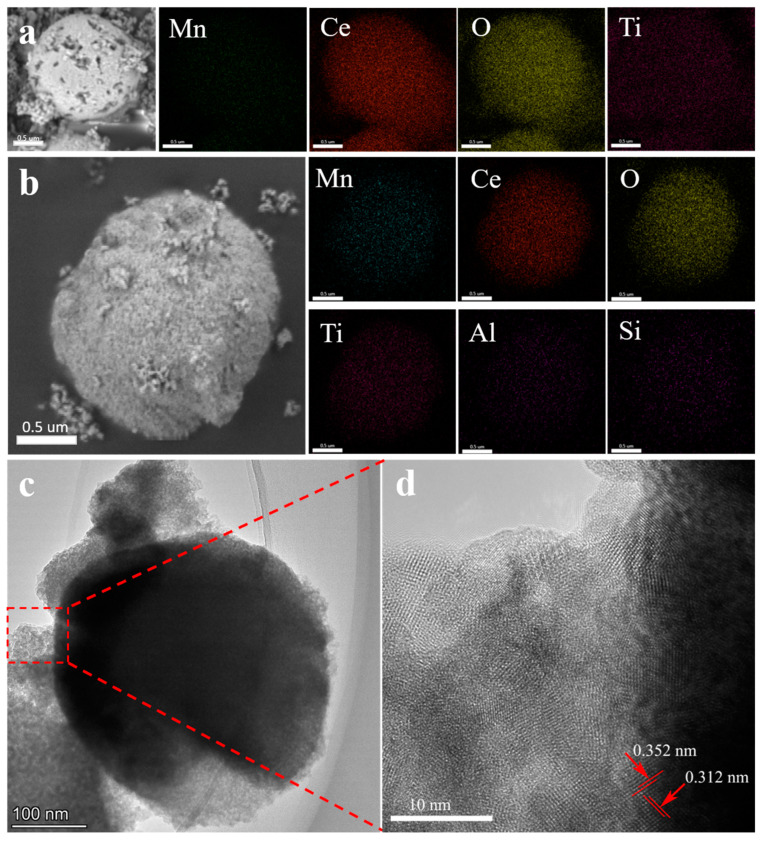
(**a**) EDS image of MnCeO*_x_*@TiO_2_ catalyst; (**b**) EDS image of MnCeO*_x_*@TiO_2_-T2 catalyst; (**c**) TEM; and (**d**) HRTEM images of MnCeO*_x_*@TiO_2_-T2 catalyst.

Figure 4a,b show the energy-dispersive spectrometer (EDS) element mapping of MnCeO*_x_*@TiO_2_ and MnCeO*_x_*@TiO_2_-T2 catalysts. It is evident that that the particle size is about 1.5 um, and TiO_2_ crystals are uniformly dopped on the spherical particle surface. In addition, the Mn, Ce, and O elements were also uniformly dispersed within the catalyst. Additionally, the uniform distribution of Al and Si elements was found on the MnCeO*_x_*@TiO_2_-T2 catalyst, which confirms the existence of tourmaline in the catalyst (the component of tourmaline is listed in Table S2). The SiO_2_ component of tourmaline can decrease the thermal stability of NH_4_HSO_4_ and thus promote its decomposition on the surface of catalyst [33]. Additionally, both SiO_2_ and Al_2_O_3_ components of tourmaline can enhance the acidity of the catalyst, weakening the adsorption of SO_2_ and thus improving the SO_2_ poisoning resistance of the catalyst [34]. Figure 4c,d showed the transmission electron microscope (TEM) and high-resolution transmission electron microscope (HRTEM) image of MnCeO*_x_*@TiO_2_-T2 catalyst, respectively. The different lattice fringes were measured to be 0.312 nm and 0.352 nm, which are well matched with the (111) crystal plane of CeO_2_ [35] and the (101) crystal plane of TiO_2_ [36], respectively.

### 2.3. Specific Surface Area and Surface Element Analysis

**Figure 5:**
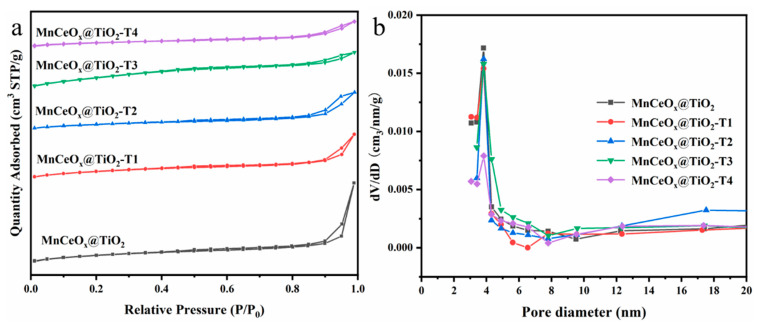
Adsorption isotherms of MnCeO*_x_*@TiO_2_ with different additions of tourmaline (**a**) and BJH pore size distribution curve (**b**).

In order to explore the effect of tourmaline addition on the specific surface area and pore of the catalyst, the N_2_ adsorption–desorption measurement was performed on the MnCeO*_x_*@TiO_2_ and MnCeO*_x_*@TiO_2_-T catalysts (Figure 5 and Table 1). All catalysts exhibited type-IV adsorption isotherms but with different types of hysteresis loops. The MnCeO*_x_*@TiO_2_, MnCeO*_x_*@TiO_2_-T1, and MnCeO*_x_*@TiO_2_-T2 catalysts correspond to the H3 type hysteresis loops [37], while the MnCeO*_x_*@TiO_2_-T3 and MnCeO*_x_*@TiO_2_-T4 catalysts follow the H4 type of hysteresis loops, both of which reflect the irregular porous structure of the catalysts. Figure 5b shows the pore size distributions of various catalysts. The pore diameters of the five catalysts are in the range of 6–13 nm, indicating that the catalysts are all mesoporous. It can be seen from Table 1 that with the increase in tourmaline content, the specific surface area of the catalyst decreases from 84.00 to 42.15 m^2^ g^–1^ gradually, which accords with the SEM result that the catalyst particles become more and more dense. However, this result is different from the reports published previously [23,24,25]. It is accepted that the micro-electric field of tourmaline would decrease the particle size of catalysts, presenting their agglomeration and thus slightly increasing the specific surface area of catalysts. In our case, this unusual phenomenon could be attributed to the blocking of the pores of MnCeO*_x_*@TiO_2_ by the impregnated species. Although the specific surface area of the catalyst was decreased, the catalytic activity of MnCeO*_x_*@TiO_2_-T2 was relatively higher. This indicates that the addition of appropriate amounts of tourmaline changed the crystal growth and preferentially exposed the active {001} facets of TiO_2_, which enhanced the catalytic activity. Additionally, the addition of tourmaline introduced the acidic SiO_2_ and Al_2_O_3_ components into the catalyst, which can improve SO_2_ poisoning resistance. We consider that these advantageous factors contribute to the improvement of the catalytic performance.

**Figure 6:**
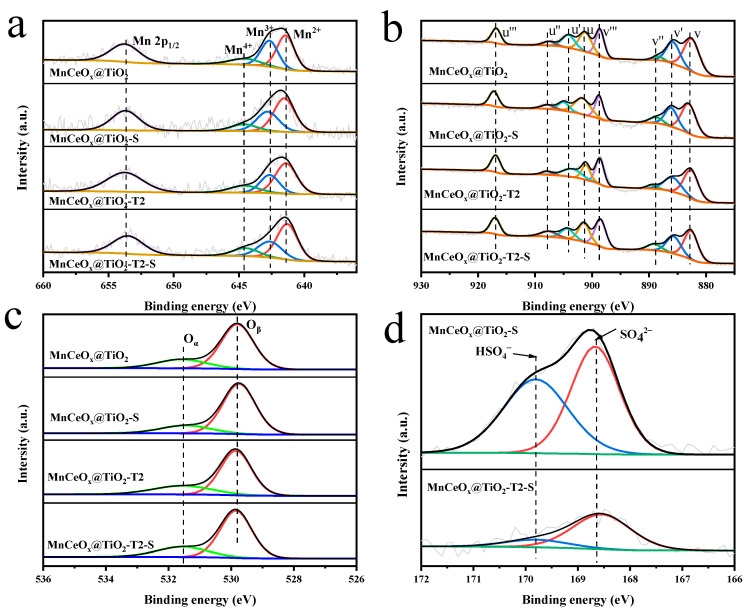
XPS diagrams of MnCeO*_x_*@TiO_2_ and MnCeO*_x_*@TiO_2_-T2 catalysts before and after poisoning (**a**) Mn2p_3/2_; (**b**) Ce3d; (**c**) O1s; and (**d**) S2p.

Figure 6 shows the X-ray photoelectron spectroscopy (XPS) spectra of Mn 2p, Ce 3d, O 1s, and S 2p of MnCeO*_x_*@TiO_2_ and MnCeO*_x_*@TiO_2_-T2 catalysts before and after catalyst poisoning. Table 2 summarizes the surface atomic concentration and relative content of Mn, Ce, O and S determined by XPS spectra. As shown in Figure 6a, the Mn 2p region is composed of spin–orbit doublets. The binding energy of Mn 2p1/2 appears about 653.7 eV, and the binding energy of Mn 2p3/2 corresponds to Mn^2+^ (641.3–641.5 eV), mixed valence states of Mn^3+^ (642.3–642.8 eV), and Mn^4+^ (644.6–643.6 eV) [38]. The relative concentration of Mn^4+^ on the surface of different tourmaline-added catalysts is different. The highest Mn^4+^ content was observed for MnCeO*_x_*@TiO_2_-T2. The redox performance of SCR is related to the surface concentration of Mn^4+^. The higher the relative concentration of Mn^4+^, the more favorable the low-temperature SCR reaction [39]. This may be another reason that MnCeO*_x_*@TiO_2_-T2 exhibited the best catalytic activity. Meanwhile, it can be found from Figure 6a that the binding energy of Mn in MnCeO*_x_*@TiO_2_-S catalyst is higher than that of the fresh catalyst, while the binding energy of Mn in MnCeO*_x_*@TiO_2_-T2-S catalyst does not change significantly compared with the fresh catalyst. This result reveals that the Mn atoms on the surface of MnCeO*_x_*@TiO_2_ bonded with electronegative S in addition to elemental O. As shown in Table 2, we can see that when the MnCeO*_x_*@TiO_2_ was poisoned by SO_2_, the content of Mn^4+^ on the surface of the catalyst decreased from 14.92% to 12.22%, while under SO_2_ atmosphere, the Mn^4+^ content of MnCeO*_x_*@TiO_2_-T2 decreased from 16.93% to 15.42%, a slighter decrease compared to MnCeO*_x_*@TiO_2_, indicating that the MnCeO*_x_*@TiO_2_ bonded with more SO_2_ than MnCeO*_x_*@TiO_2_-T2. That is, MnCeO*_x_*@TiO_2_-T2 has better SO_2_-tolerance performance.

The XPS spectra of Ce 3d in catalysts can be fitted into eight peaks, as shown in Figure 6b. Ce^3+^ is shown by the subbands labeled u′ and v′, and Ce^4+^ is shown by the subbands labeled u, u″, u‴, v, v″, and v‴ [40]. The slight shift in the Ce 3d spectra to higher binding energies was observed in MnCeO*_x_*@TiO_2_ when the catalyst was introduced to SO_2_, suggesting that the density of electron clouds of Ce in MnCeO*_x_*@TiO_2_ was decreased due to oxidation by O_2_. However, no obvious change in binding energies of Ce 3d was found for MnCeO*_x_*@TiO_2_-T2, revealing that MnCeO*_x_*@TiO_2_-T2 had outstanding SO_2_-tolerance performance [41]. Figure 6c shows the O 1s spectra of MnCeO*_x_*@TiO_2_ and MnCeO*_x_*@TiO_2_-T2 before and after SO_2_ poisoning. The O 1s spectra can be divided into two peaks. The band at high binding energy (531.5 eV) is attributed to chemically adsorbed oxygen, denoted by O_α_. The low binding energy band (529.8 eV) corresponds to lattice oxygen, denoted as O_β_ [42]. NH_3_-SCR is a gas–solid reaction, and the surface-active oxygen plays a key role in catalyzing NH_3_-SCR reaction. Due to its higher mobility, the performance of chemically adsorbed oxygen in the surface oxidation reaction is better than that of lattice oxygen [43]. It can be seen in Table 2 that the relative content of O_α_ in the MnCeO*_x_*@TiO_2_-T2 catalyst is 26.16%, which is slightly higher than that of other catalysts, indicating that the introduction of a small amount of tourmaline into MnCeO*_x_*@TiO_2_ increased the O_α_ content. We can also see from Table 2 that the O_α_ content of the poisoned MnCeO*_x_*@TiO_2_-T2-S catalyst is slightly reduced to 25.98%, which is much higher than the O_α_ content (20.38%) of the MnCeO*_x_*@TiO_2_-S catalyst. It is well known that O_α_ species are beneficial to the oxidation of NO from NO to NO_2_, which promotes the NH_3_-SCR reaction through the “fast SCR” approach [44,45]. Therefore, it is proved that the addition of appropriate amount of tourmaline can significantly improve the catalytic activity and anti-poisoning performance of the catalyst. Figure 6d shows the S 2p spectra of MnCeO*_x_*@TiO_2_-S and MnCeO*_x_*@TiO_2_-T2-S. Two peaks at 169.8 eV and 168.5 eV were observed, which are attributed to HSO_4_^−^ and SO_4_^2−^ [46,47], respectively, indicating the presence of sulfate and bisulfate on the catalyst surface, and the catalysts were sulfated to generate ammonium salt, manganese salt and cerium salt. Compared to the MnCeO*_x_*@TiO_2_-S catalyst, the content of HSO_4_^−^ and SO_4_^2−^ on the surface of the MnCeO*_x_*@TiO_2_-T2-S catalyst is much less, indicating that the MnCeO*_x_*@TiO_2_-T2 catalyst is not easy to be sulfated and effectively inhibits the deposition of NH_4_HSO_4_ on the surface of the catalyst. As shown in Table 2, the relative concentration of Mn^4+^ and Ce^3+^ ions on the surface of the poisoned catalyst decreased, while the relative concentration of Mn^2+^ and Ce^4+^ ions increased and meanwhile, the binding energy of Mn^2+^ and Ce^4+^ increased, both of which indicate that MnSO_4_ and Ce(SO_4_)_2_ were formed, which led to the deactivation of catalysts [48,49,50].

### 2.4. Redox Capability

**Figure 7:**
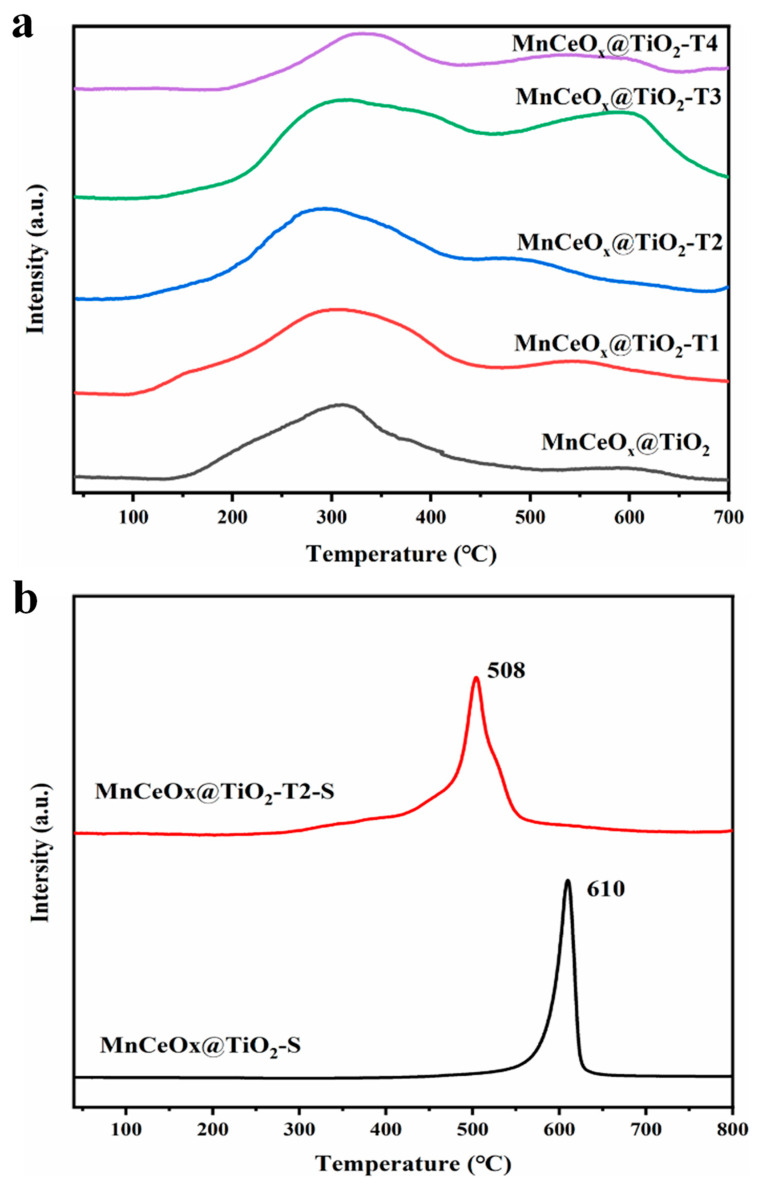
(**a**) H_2_-TPR diagrams of MnCeO*_x_*@TiO_2_ with different additions of tourmaline. (**b**) H_2_-TPR diagram of MnCeO*_x_*@TiO_2_-S and MnCeO*_x_*@TiO_2_-T2-S catalysts after poisoning.

The H_2_-TPR diagram of the catalysts is shown in Figure 7. The reduction peaks of the catalysts are superimposed. The H_2_ consumption peak below 450 °C is attributed to the reduction of MnO_2_→Mn_2_O_3_ and Mn_2_O_3_→Mn_3_O_4_ and the reduction of surface cerium. At the same time, the peak at about 600 °C is the reduction of bulk cerium. The addition of appropriate amount of tourmaline (1%–3%) caused the H_2_ consumption peak of the catalyst to shift to low temperature (Figure 7a), indicating that the reduction temperature of H_2_ was low and the redox performance of catalysts was strong [49]. However, the addition of excess amount of tourmaline (4%) caused an increase in H_2_ reduction temperature (Figure 7a), which is in accordance with the catalytic performance result. It can be deduced that the addition of appropriate amounts of tourmaline promoted the enhancement of the oxidation ability of Mn species and active components on the catalyst surface and thus improved the low-temperature SCR reaction [50].

The hydrogen-temperature-programmed reduction (H_2_-TPR) diagram of the SO_2_ poisoned catalysts is shown in Figure 7b. The reduction peaks of MnCeO*_x_*@TiO_2_-S and MnCeO*_x_*@TiO_2_-T2-S catalysts appeared at 610 and 508 °C, respectively and are attributed to the reduction in MnSO_4_ [51]. The presence of MnSO_4_ confirmed the sulfation of active ingredient manganese oxide on the surface of the catalyst. However, compared to the MnCeO*_x_*@TiO_2_-S catalyst, the reduction peak temperature of the MnCeO*_x_*@TiO_2_-T2-S catalyst shifted to low temperature, indicating that the oxidation–reduction performance of the MnCeO*_x_*@TiO_2_-T2 catalyst was better than MnCeO*_x_*@TiO_2_ after SO_2_ poisoning. The results show that under high SO_2_ concentration, the reduction ability of the poisoned catalyst is weakened due to the decrease in the relative concentration of Mn^4+^, which is consistent with the XPS results.

### 2.5. Study on the Mechanism of Catalyst Anti-Poisoning Reaction

#### 2.5.1. Adsorption of SO_2_ + O_2_ on the Catalyst Surface

**Figure 8:**
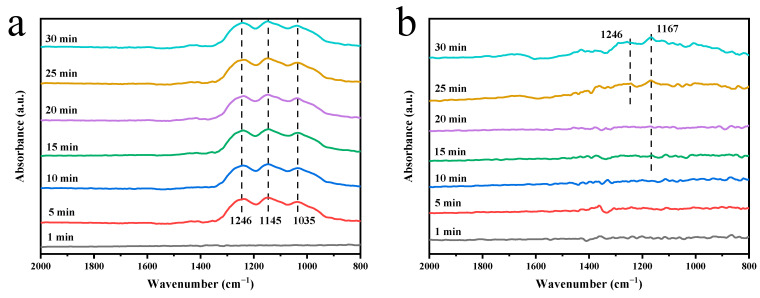
In situ DRIFTS spectra of SO_2_+O_2_ adsorption on the surface of catalyst (**a**) MnCeO*_x_*@TiO_2;_ (**b**) MnCeO*_x_*@TiO_2_-T2.

In order to analyze the adsorption behavior of SO_2_ on the surface of MnCeO*_x_*@TiO_2_ and MnCeO*_x_*@TiO_2_-T2 catalysts, in situ diffuse reflectance infrared Fourier transform spectroscopy (In situ DRIFT) was carried out, as shown in Figure 8. We introduced 500 ppm SO_2_ + 5 vol.% O_2_ gas to the surfaces of the two catalysts at 170 °C. After the introduction of SO_2_ to MnCeO*_x_*@TiO_2_ for 5 min, several peaks were detected at 1246, 1145, and 1035 cm^–1^ (Figure 8a). These peaks can be attributed to the sulfate species formed on the catalyst surface [52,53]. It can be thus deduced that the MnCeO*_x_*@TiO_2_ catalyst was strongly sulfated, and the active sites of the SCR reaction were occupied by the sulfate species adsorbed on the catalyst surface, which will affect the adsorption and activation of NH_3_ and NO*_x_* species on the catalyst surface, inhibiting the SCR reaction progress. Figure 8b shows the in situ DRIFTS spectra of the MnCeO*_x_*@TiO_2_-T2 catalyst after adsorbing 500 ppm SO_2_ + 5 vol.% O_2_. Only 15 min after the introduction of SO_2_, the faint infrared peak attributed to the surface sulfate species (1167 cm^−1^) was detected. With continuous introduction of SO_2_ for 25 min, the peaks at 1246 and 1167 cm^−1^ attributable to the surface sulfate species gradually increased. The surface sulfate species of the MnCeO*_x_*@TiO_2_-T2 catalyst are much less than that of the MnCeO*_x_*@TiO_2_ catalyst, and the prolonged time of resistance to SO_2_ poisoning indicates that the addition of appropriate amount of tourmaline can effectively improve the anti-poisoning performance of the catalyst.

#### 2.5.2. Adsorption of NH_3_ + SO_2_ on the Catalyst Surface

**Figure 9:**
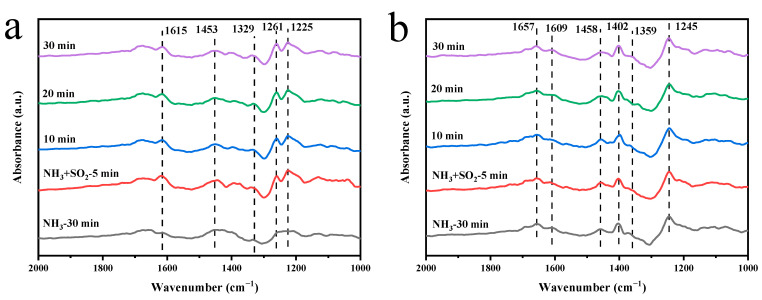
In situ DRIFTS spectra of (**a**) MnCeO*_x_*@TiO_2_ and (**b**) MnCeO*_x_*@TiO_2_-T2 upon exposure to the NH_3_ + SO_2_.

As shown in Figure 9a, when the MnCeO*_x_*@TiO_2_ catalyst was exposed to NH_3_ for 30 min, new adsorption bands appeared at 1615, 1453 and 1329 cm^–1^. The bands at 1615 and 1170 cm^−1^ are attributable to the coordinated NH_3_ at the Lewis acid site [54,55], and the band at 1453 cm^−1^ is attributable to the NH_4_^+^ symmetric bending vibration at the Brønsted acid site [56]. It can be found that after adding 100 ppm SO_2_ for 5 min, the adsorption peak intensity corresponding to the coordinated NH_3_ on the Lewis acid site and the NH_4_^+^ formed on the Brønsted acid site has increased, but due to the continuous introduction of SO_2_, after 10 min, the peak intensity at 1615 and 1453 cm^−1^ gradually weakened. However, two adsorption bands appeared at 1261 and 1225 cm^−1^ after the introduction of SO_2_, which was attributed to SO_2_ adsorption. The results show that SO_2_ and NH_3_ are competitively adsorbed on the surface of MnCeO*_x_*@TiO_2_ catalyst. The addition of SO_2_ occupied some adsorption sites of NH_3_ on the catalyst surface, thereby inhibiting the adsorption and activation of NH_3_.

Figure 9b shows the in situ DRIFTS spectra of the MnCeO*_x_*@TiO_2_-T2 catalyst upon exposure to the NH_3_ + SO_2_ atmosphere. Thirty minutes after the introduction of only NH_3_, several obvious adsorption bands were detected at 1657, 1609, 1458, 1402, 1359, and 1245 cm^−1^. The bands at 1609 and 1245 cm^–1^ are assigned to the coordinated NH_3_ on the Lewis acid site, and the band at 1359 cm^–1^ is attributed to the amino compound (-NH_2_) on the catalyst surface [57]. The peaks around 1657, 1458, and 1402 cm^−1^ are attributed to the symmetrical bending vibration of the coordinated NH_4_^+^ adsorbed on the Brønsted acid site by NH_3_. In the presence of 100 ppm SO_2_, the intensity of the adsorption zone is basically unchanged. The addition of SO_2_ caused the occupation of part of the catalytic adsorption sites on the surface of the MnCeO*_x_*@TiO_2_ catalyst by SO_2_, thereby inhibiting the adsorption and activation of NH_3_. In contrast, the intensity of the adsorption peak of NH_3_ species adsorbed on the MnCeO*_x_*@TiO_2_-T2 catalyst did not decrease. The results show that the NH_3_ adsorption capacity of the MnCeO*_x_*@TiO_2_-T2 catalyst is stronger than that of the MnCeO*_x_*@TiO_2_ catalyst. In this case, SO_2_ did not significantly inhibit the adsorption of NH_3_. This confirms that the addition of appropriate amounts of tourmaline effectively prevents the adsorption of SO_2_ on the surface of the catalyst. The added tourmaline can increase the number of Brønsted acid sites. The strong electronic interactions between HSO_4_^−^ and Brønsted acid sites of mesoporous TiO_2_ damaged the bond between NH_4_^+^ and HSO_4_^−^, thus promoting the decomposition of NH_4_HSO_4_ and improving the catalytic activity and the anti-SO_2_ performance.

#### 2.5.3. Adsorption of NO + O_2_ + SO_2_ on the Catalyst Surface

**Figure 10:**
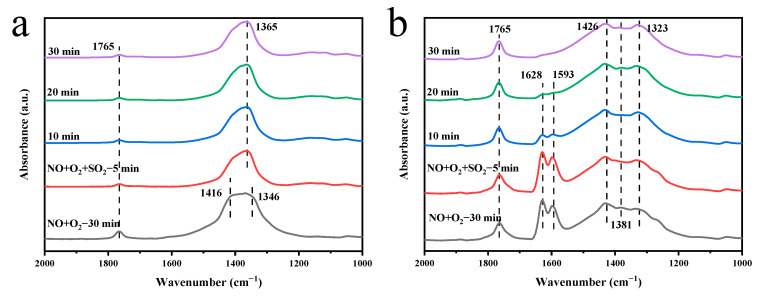
Effect of SO_2_ on the adsorption of NO + O_2_ on the surface of (**a**) MnCeO*_x_*@TiO_2_ and (**b**) MnCeO*_x_*@TiO_2_-T2 by in situ DRIFTS spectra.

The effect of SO_2_ on the adsorption of NO + O_2_ on the MnCeO*_x_*@TiO_2_ catalyst was also studied by in situ DRIFTS, and the results are shown in Figure 10a. The peak at 1765 cm^−1^ is attributed to the N_2_O_4_ species [58], while the peak at 1416 cm^−1^ is the infrared peak of nitrate, and the peak at 1346 cm^−1^ is attributed to bidentate nitrate [59]. When the catalyst was exposed to 100 ppm SO_2_, the peaks at 1416 and 1346 cm^−1^ disappeared and a new peak appeared at 1365 cm^−1^, which can be attributed to the asymmetric tensile vibration of O=S=O in the sulfate component on the catalyst surface [60]. These results indicate that SO_2_ and NO have competitive adsorption on the catalyst surface and that the adsorption capacity of SO_2_ on the catalyst surface is significantly stronger than that of NO. The effect of SO_2_ on the adsorption of NO + O_2_ on the MnCeO*_x_*@TiO_2_-T2 catalyst is shown in Figure 10b. After the exposure to NO + O_2_ for 30 min, the peak at 1765 cm^−1^ is attributed to the N_2_O_4_ species [58], the peak at 1628 cm^−1^ is attributed to bridging nitrate [61], the peak at 1593 cm^−1^ is the infrared signal of nitrite [62], the peak at 1426 cm^−1^ is due to the linear nitrite species, and the peaks at 1381 and 1323 cm^−1^ are due to the infrared signals of bidentate nitrate [63]. There is also a competitive adsorption of NO and SO_2_ on the surface of the MnCeO*_x_*@TiO_2_-T2 catalyst. It should be pointed out that the bridging nitrate (1628 cm^−1^) and nitrite (1593 cm^−1^) peaks disappeared when SO_2_ was introduced for 20 min, while the bands of other nitrate species still exist stably. However, the peak of the nitrate species of MnCeO*_x_*@TiO_2_ catalyst disappeared after the introduction of SO_2_ for 5 min, suggesting that the adsorption capacity of MnCeO*_x_*@TiO_2_-T2 catalyst for NO is also stronger than that of the MnCeO*_x_*@TiO_2_ catalyst.

#### 2.5.4. Adsorption of NH_3_ on the Catalyst Surface before and after Poisoning

**Figure 11:**
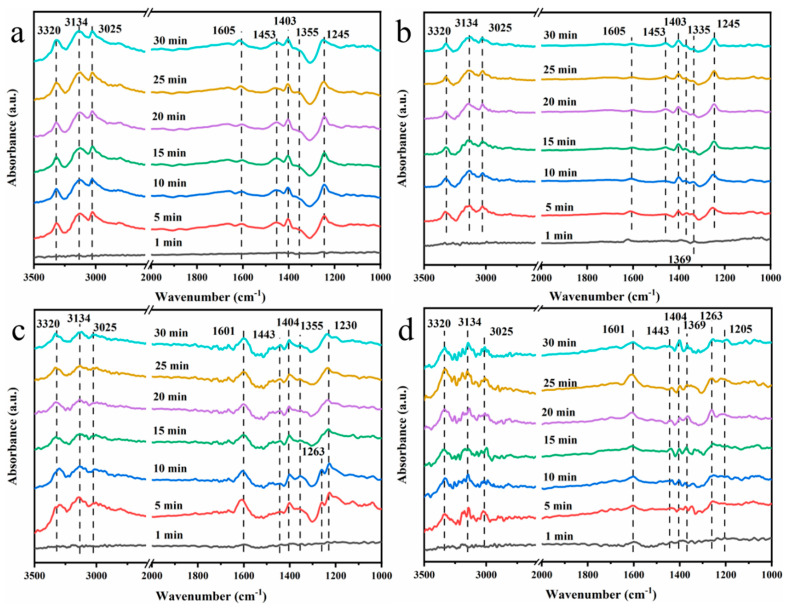
Adsorption of NH_3_ on the surface of MnCeO*_x_*@TiO_2_ catalyst (**a**) before poisoning; (**b**) after poisoning. Adsorption of NH_3_ on the surface of MnCeO*_x_*@TiO_2_-T2 catalyst (**c**) before poisoning; (**d**) after poisoning.

The in situ DRIFTS spectra of NH_3_ adsorbed on the surface of MnCeO*_x_*@TiO_2_ and MnCeO*_x_*@TiO_2_-2 catalyst before and after poisoning for different time are shown in Figure 11. Compared to the non-poisoned catalyst, the symmetrical and asymmetrical stretching vibration peaks (3320, 3134, and 3025 cm^−1^) of the N-H bond in the coordination state of NH_3_ did not change significantly [64]. The peak of NH_3_ coordinated on the Lewis acid (1605 and 1245 cm^−1^) and the coordinated NH_4_^+^ peaks (1403 and 1453 cm^−1^) attributable to the Brønsted acid site of the poisoned MnCeO*_x_*@TiO_2_ catalyst were significantly weakened. The 1355 cm^−1^ peak attributable to the oxidized and deformed intermediate generated by the adsorption of NH_3_ on the Lewis acid site migrates to 1369 cm^−1^ [65], and a new weak peak appears at 1335 cm^–1^, which is attributed to the adsorption coordination of the NH_3_ peak on the Lewis acid. This is because the presence of SO_2_ and H_2_O will weaken the Brønsted acid sites on the MnCeO*_x_*@TiO_2_ catalyst, thereby weakening the adsorption of NH_3_.

The in situ DRIFTS spectra of the MnCeO*_x_*@TiO_2_-T2 catalyst before and after the poisoning of NH_3_ adsorption for different time are shown in Figure 11c,d. Compared tothe non-poisoned catalyst, the peak of NH_3_ coordinated on the Lewis acid (1605 and 1245 cm^−1^) of the poisoned MnCeO*_x_*@TiO_2_-T2 catalyst are significantly weakened. The peak at 1355 cm^−1^, which is attributed to the oxidation and deformation intermediates generated by the adsorption of NH_3_ on the Lewis acid site, shifts to 1369 cm^–1^. A new peak around 1205 cm^−1^ appeared, which is attributable to the peak of NH_3_ adsorbed on the Lewis acid, while the peak attributed to the Brønsted acid site has no obvious change, which means that in the presence of SO_2_ and H_2_O, the MnCeO*_x_*@TiO_2_-T2 catalyst inhibits the loss of Brønsted acid sites and effectively inhibits the bonding of NH_4_^+^ and HSO_4_^−^, and meanwhile promotes the decomposition of NH_4_HSO_4_, thus improving the anti-poisoning performance of the catalyst.

#### 2.5.5. Adsorption of NO + O_2_ on the Catalyst Surface before and after Poisoning

**Figure 12:**
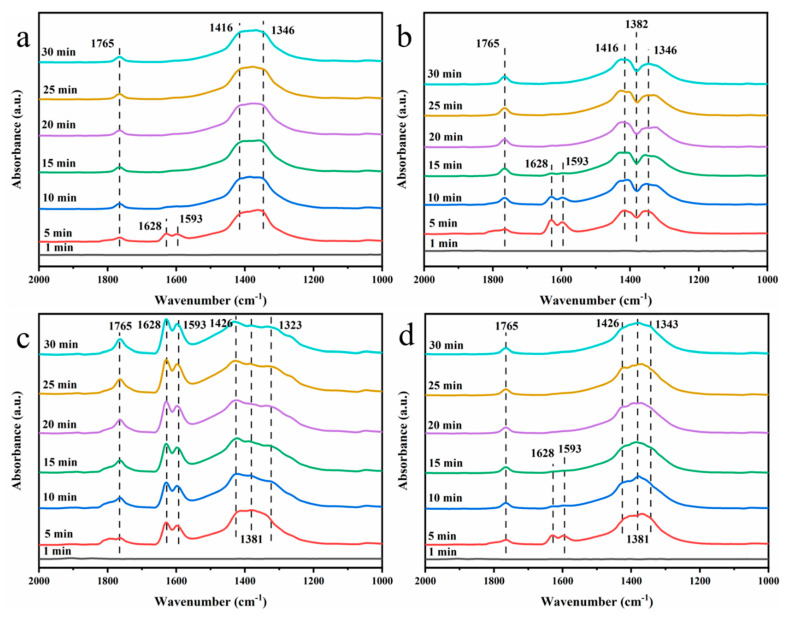
Adsorption of NO + O_2_ on the surface of MnCeO*_x_*@TiO_2_ catalyst (**a**) before poisoning; (**b**) after poisoning. Adsorption of NO + O_2_ on the surface of MnCeO*_x_*@TiO_2_-T2catalyst (**c**) before poisoning; (**d**) after poisoning.

The in situ DRIFTS spectra of NO + O_2_ adsorbed on the MnCeO*_x_*@TiO_2_ catalyst before and after poisoning are shown in Figure 12a,b. The peak at 1765 cm^−1^ is attributed to the N_2_O_4_ species, the peak at 1628 cm^−1^ is attributed to bridged nitrate, 1593 cm^−1^ is the infrared absorption peak of nitrite, 1416 cm^−1^ is the infrared peak of nitrate, and the peak at 1346 cm^−1^ is attributed to bidentate nitrate. Compared to the non-poisoned catalyst, the poisoned MnCeO*_x_*@TiO_2_ catalyst bridges the peaks of nitrate (1628 cm^−1^) and nitrite (1593 cm^−1^) only after 15 min, which is due to the fact that after H_2_O and SO_2_ poisoning, ammonium sulfate was generated that cover the active sites on the catalyst surface, thereby inhibiting the adsorption of NO on the catalyst surface. The peak at 1382 cm^−1^ is due to the gas adsorbed on the surface of the catalyst, SO_2_ and H_2_O are poisoned to form an asymmetric tensile vibration of O=S=O in the surface sulfate composition, NO at O=S=O after adsorption, O=S=O was shielded, so a negative peak was generated at 1382 cm^–1^ [66,67]. The in situ DRIFTS spectra of NO + O_2_ adsorbed on the MnCeO*_x_*@TiO_2_-T2 catalyst before and after poisoning are shown in Figure 12c,d. The bidentate nitrate peak at 1323 cm^−1^ disappeared on the poisoned catalyst, and the peak bridging the nitrate (1628 cm^−1^) and the nitrite peak (1593 cm^−1^) also disappeared after 15 min, and a new bidentate nitrate peak appeared at 1343 cm^−1^, which may be due to the MnCeO*_x_*@TiO_2_-T2 catalyst undergoing H_2_O and after SO_2_ is poisoned, the produced sulfate or ammonium sulfate will have new adsorption sites for NO. It shows that even if the MnCeO*_x_*@TiO_2_-T2 catalyst is affected by the poisoning of SO_2_ and H_2_O, the adsorption of NO at some active sites is inhibited, but the MnCeO*_x_*@TiO_2_-T2 catalyst can still generate new adsorption sites for NO and formed bidentate nitrate at this site.

#### 2.5.6. Anti-Poisoning Mechanism of MnCeO_x_@TiO_2_-T2 Catalyst

**Figure 13:**
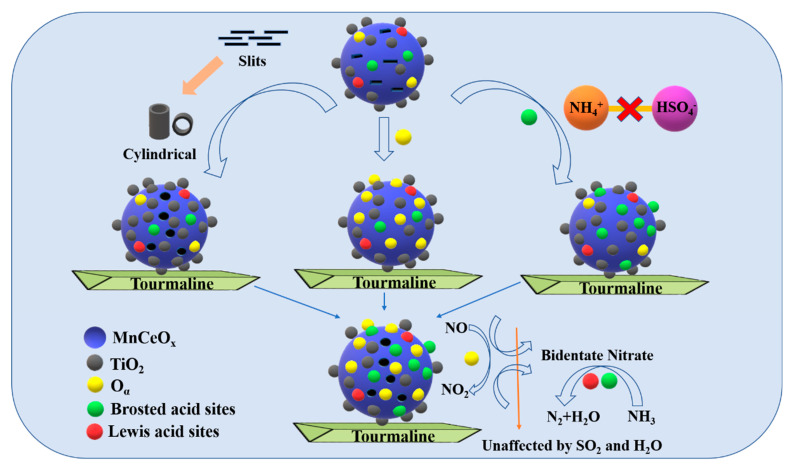
Schematic diagram of the anti-poisoning mechanism of MnCeO*_x_*@TiO_2_-T2 catalyst.

The schematic diagram of the anti-poisoning mechanism of the MnCeO*_x_*@TiO_2_-T2 catalyst is shown in Figure 13. The addition of appropriate amounts of tourmaline effectively exposes the active {001} facets and meanwhile introduces the acidic SiO_2_ and Al_2_O_3_ components into the catalyst that can improve the catalytic activity and enhance the anti-poisoning performance. In addition, the addition of tourmaline reduces the adsorption of SO_2_ on the catalyst surface and the competitive adsorption of NH_3_ and NO by SO_2_. Compared to the MnCeO*_x_*@TiO_2_ catalyst, the MnCeO*_x_*@TiO_2_-T2 catalyst has stronger NH_3_ and NO adsorption capacity. Additionally, MnCeO*_x_*@TiO_2_-T2 exhibits more Brønsted acid sites and thus weakens the SO_2_ adsorption. As a result, MnCeO*_x_*@TiO_2_-T2 efficiently inhibited the deposition of NH_4_HSO_4_ and (NH_4_)_2_HSO_4_ on the surface of the catalyst, thereby improving the NH_3_-SCR performance and anti-poisoning ability of the catalyst.

## 3. Experimental Section

### 3.1. Preparation of Catalysts

Manganese acetate and cerium nitrate (in the molar ratio 4:6) were added to a mixture of ethylene glycol (30 mL) and isopropanol (30 mL). The mixture was ultrasonically stirred until dissolved. The solution was transferred in a 100 mL hydrothermal kettle (lined with polytetrafluoroethylene) and heated at 180 °C for 24 h. The solid was filtered and washed with absolute ethanol and deionized water three times. After drying at 80 °C for 6 h, the product was obtained and designated as MnCeO*_x_*. After that, 0.52 g of MnCeO*_x_* was dissolved in 200 mL of absolute ethanol through ultrasonic treatment for 30 min. 0.52 g of urea was then added to the above solution under continuous sonication for another 30 min. 1.5 mL of butyl titanate was dispersed in 20 mL of ethanol and the resulting dispersion was added dropwise to the above solution. The mixture was stirred at 45 °C for 24 h. The obtained precipitate was filtrated and washed with deionized water several times and then dried at 80 °C for 12 h. Finally, the sample was calcined in a muffle furnace at 500 °C for 4 h (heating rate 1 °C min^−1^) to obtain MnCeO*_x_*@TiO_2_. To investigate the effect of tourmaline on the denitration performance, a suspension of butyl titanate (1.5 mL) and a certain amount of tourmaline in 20 mL of ethanol was used instead, and the other procedure was the same, with the preparation of MnCeO*_x_*@TiO_2_. The mass ratios of tourmaline and MnCeO*_x_*@TiO_2_ are 1%, 2%, 3%, and 4%, respectively, and the tourmaline-doped MnCeO*_x_*@TiO_2_ is denoted as MnCeO*_x_*@TiO_2_-T1, MnCeO*_x_*@TiO_2_-T2, MnCeO*_x_*@TiO_2_-T3, and MnCeO*_x_*@TiO_2_-T4, respectively. After SO_2_ and H_2_O poisoning, MnCeO*_x_*@TiO_2_ and MnCeO*_x_*@TiO_2_-T catalysts are denoted as MnCeO*_x_*@TiO_2_-S and MnCeO*_x_*@TiO_2_-T-S, respectively.

### 3.2. Catalytic Performance Tests

The NH_3_-SCR performance testing was carried out in a fixed-bed quartz reactor (inner diameter of 8 mm). The typical reaction condition is as follows: [NO] = [NH_3_] = 500 ppm, [O_2_] = 5 vol.%, and N_2_ balance; 0.5 mL of catalyst (40–60 mesh) was used for the SCR activity study. The total flow rate of feed gases was 100 mL min^−1^, and the GHSV was 10,000 h^−1^. KM940 flue (UK, KANE) gas analyzer was used to analyze the feed gases and the effluent streams. The reaction temperature ranged from 100 to 450 °C. The NO*_x_* conversion was calculated according to Equation (1):

(1) $$x=\frac{{\left[{NO}_{x}\right]}_{in}-{\left[{NO}_{x}\right]}_{out}}{{\left[{NO}_{x}\right]}_{in}}\times 100\%$$

where [NO*_x_*]_in_ and [NO*_x_*]_out_ are the inlet and outlet concentration at steady-state, respectively.

## 4. Conclusions

In summary, a series of MnO*_x_*/CeO_2_ bimetallic oxide catalysts were synthesized, and mesoporous TiO_2_ was doped on the surface of the MnO*_x_*/CeO_2_ with the addition of different amounts of tourmaline. The effect of tourmaline on the NH_3_-SCR catalytic performance of MnCeO*_x_*@TiO_2_ was intensively investigated. The catalytic experiments reveal that the addition of appropriate amounts of tourmaline (2 wt%) can improve the low-temperature denitration performance of the catalyst. The MnCeO*_x_*@TiO_2_-T2 catalyst exhibited the most excellent NH_3_-SCR activity, and the NO*_x_* conversion is maintained at 100% in the temperature window of 110–200 °C. After the exposure to 300 ppm SO_2_ and 10 vol.% H_2_O at 170 °C, the denitration activity of the catalyst can still be maintained above 90%. Although the specific surface area of catalysts was decreased with the addition of tourmaline, the active {001} facets of TiO_2_ were more exposed and the content of Mn^4+^ and O_α_ on the surface of the catalyst was increased. In addition, the addition of tourmaline introduced the acidic SiO_2_ and Al_2_O_3_ components that is favorable for the improvement of catalytic activity. The in situ DRIFTS measurement indicated that the addition of appropriate amounts of tourmaline increased the number of Brønsted acid sites on the catalyst surface and weakened the adsorption of SO_2_, suppressing the deposition of ammonium sulfate on the surface of the catalyst, resulting in the enhanced NH_3_-SCR performance and anti-poisoning ability of the catalyst.

## Figures and Tables

**Table 1:** The specific surface area, pore volume and average pore diameter of MnCeO*_x_*@TiO_2_ with different additions of tourmaline.

Catalyst	Specific Surface Area (m^2^ g^–1^)	Pore Volume (cm^3^ g^–1^)	Pore Size (nm)
MnCeO*_x_*@TiO_2_	84.00	0.14	6.70
MnCeO*_x_*@TiO_2_-T1	81.55	0.16	7.73
MnCeO*_x_*@TiO_2_-T2	66.77	0.14	8.17
MnCeO*_x_*@TiO_2_-T3	56.66	0.14	9.64
MnCeO*_x_*@TiO_2_-T4	42.15	0.15	12.81

**Table 2:** The content and relative concentration of surface elements before and after poisoning of MnCeO*_x_*@TiO_2_ and MnCeO*_x_*@TiO_2_-T2.

Catalyst	Surface Atom Concentration (%)					Relative Concentration (%)			
	Mn	Ce	Ti	O	S	Mn^4+^	Mn^2+^	Ce^3+^	O_α_
MnCeO*_x_*@TiO_2_	2.32	2.80	17.04	51.27	-	7.46	24.37	13.93	23.07
MnCeO*_x_*@TiO_2_-S	1.35	2.39	15.85	54.95	5.25	6.11	26.73	11.28	20.38
MnCeO*_x_*@TiO_2_-T2	2.26	2.96	18.06	53.12	-	8.46	27.18	14.25	26.16
MnCeO*_x_*@TiO_2_-T2-S	2.20	2.89	18.76	54.01	1.18	7.71	27.81	11.52	25.98

## Data Availability

The authors confirm that most of the data supporting the findings of this study are available within the article and its supplementary material. Raw data are available from the corresponding author (Y.G.) on request.

## Supplementary Materials

The following supporting information can be downloaded at: https://www.mdpi.com/article/10.3390/molecules29174079/s1, Figure S1: PXRD patterns of MnCeO*_x_*@TiO_2_ with the addition of different amounts of tourmaline. Table S1: Comparison of the catalytic performance with previous work [14,68,69,70,71,72,73,74,75,76,77,78,79,80,81,82,83,84]. Table S2: Main composition of tourmaline (wt. %).
