# Supplementary material for: Effect of Tourmaline Addition on the Anti-Poisoning Performance of MnCeOx@TiO2 Catalyst for Low-Temperature Selective Catalytic Reduction of NOx
Source: Molecules. 2024 Aug 28;29(17):4079. doi: 10.3390/molecules29174079 (PMC11396665; doi:10.3390/molecules29174079)
Supplement: Supplementary file 1 [file molecules-29-04079-s001.zip › molecules-3165658-supplementary.pdf]

Supporting Information

Effect of Tourmaline Addition on the Anti-poisoning Performance of $MnCeO_x$@TiO_2 Catalyst for Low-Temperature Selective Catalytic Reduction of NO_x

Zhenzhen Zhao,[a,#] Liyin Wang,[b,#] Qingxiang Lin,[a] Gang Xue,[b,*] Hui Hu,[c] Haibin Ma,[d] Ziyu Wang,[a] Xiaofang Su,[c] and Yanan Gao[c,*]

[a] School of Advanced Agricultural Science, Weifang University, Weifang, 261061, China;

[b] Institute of Power Source and Ecomaterials Science, Hebei University of Technology, Tianjin 300130, China;

[c] Key Laboratory of Ministry of Education for Advanced Materials in Tropical Island Resources, Hainan University, Haikou 570228, China;

[d] School of Chemistry, Chemical & Environmental Engineering, Weifang University, Weifang, 261061, China.

Corresponding author: xuegang@hebut.edu.cn (G. Xue); ygao@hainanu.edu.cn (Y. Gao).

Materials

Manganese Acetate, butyl titanate, cerium nitrate, ethylene glycol, tourmaline and isopropyl alcohol were purchased from Tianjin Damao Chemical Reagent Factory. Urea and anhydrous ethanol were obtained from Tianjin Fenfan Technology Development Co., Ltd. All gases, including N_2 (99.999%), O_2 (99.999%), NH_3 (10 vol. % in N_2), NO (10 vol. % in N_2) and SO_2 (10 vol. % in N_2) were purchased from Tianjin Vista gas Co. Ltd.

Catalyst Characterization

Fourier-transform infrared (FT-IR) spectra were recorded on a JASCO/ FTIR-6800. The nitrogen adsorption and desorption isotherms were measured at 77 K using a Autosorb-IQ2-C-TPX. The elements in the material were determined using an X ray energy

spectrum analysis (EDAX, Thermo SCIENTIFIC Nexsa). Powder X-ray diffraction (XRD) measurement was used to analyze the crystal structure of the catalysts and the supports. It was performed on an X'pert3 Powder X-ray diffractometer (PANalytical, Inc., Netherlands) with an angle of 10° to 90°. The X ray source is a copper (Cu) target with a voltage of 40 kV and a current of 40 mA. SEM images of the catalysts were recorded on Sigma 300 (ZEISS, Germany) and TEM images of the samples were recorded on Tecnai G2 20 (FEI Corp., Netherlands). The specific surface area and pore volume of the catalysts were determined by N_2 absorption at −196 °C using a Micromeritics ASAP2020 system and they were calculated respectively by the Brunauer-Emmett-Teller (BET) and Barrett-Joyner-Halend (BJH) methods. Temperature programmed desorption of NH_3 (NH_3-TPD) experiments were carried out to study the acidity properties of the catalysts on a chemisorption analyser (Micromeritics, AutoChem II2920) using 0.1 g samples. Each sample was pretreated at 500 °C in He (20 mL/min) for 0.5 h. After cooled to 100 °C, the samples were exposed to pure NH_3 atmosphere for 30 min and then cooled to 50 °C, followed by purging in He for 1 h. When the baseline remained unchanged, the TPD profile of the catalysts was recorded by a TCD detector as the sample was heated from 50 to 650 °C in a flow of He (10 mL/min) with a temperature ramp of 10 °C/min. The total concentrations of various elements (Mn and Ce) in each catalyst sample were obtained by inductively coupled plasma optical emission spectrometry (ICP-OES) analysis, which was performed on an Agilent 5110 (Agilent, America). Characterization of the valences of the surface elements and the atomic surface concentrations of the catalysts was examined using X-ray photoelectron spectroscopy (XPS) (AXISULTRA DLD-600W, Shimadzu Kratos Corporation, Japan). During the measurement, the normal operating pressure used in the analysis was 10−9 Pa. The steps for analyzing and testing the sample followed standard operating procedures and the two catalysts performed the same treatment steps. The H_2 TPR experiments were performed using an Autosorb-IQ2-C-TPX which was retrofitted with a Fisons Sensorlab 200D mass spectrometer. The catalyst dosage was 20 mg. Firstly, the sample was pretreated at 300 ° C for 2 hours under N_2 atmosphere, then reduced to 40 °C. The gas is switched to a 10% H_2/N_2 mixture, and the change of H_2 is recorded using a thermal conductivity detector (TCD). The test temperature is 40-800 °C, and the heating rate is 10 °C/min. X-ray photoelectron spectroscopy (XPS) analysis was used to analyze the chemical elemental composition of the catalyst surface. Use the ESCALAB 250Xi X-ray photoelectron spectroscopy tester produced by Thermo Fisher Scientific in the United States for XPS

testing. When analyzing, the C1s peak at 284.8 eV was used as the calibration standard for energy correction, and the influence of charge effect was deducted. XPS PEAK41 software was used for Lorentzian Gaussian fitting. In situ diffuse reflectance infrared Fourier transform spectroscopy (in situ DRIFTS, TENSOR II) was used to analyze the surface adsorption and desorption, active species, and active centers of the catalyst, and to speculate on the catalytic reaction mechanism and anti-poisoning mechanism. The catalyst anti poisoning performance test is conducted on a denitrification activity evaluation device. The testing method is to stabilize at 170 ° C for 1 hour, then introduce 100-300 ppm SO_2, run for 36 hours, then introduce 10% H_2O (volume percentage), continue running for 24 hours, and stop introducing. Record the NO_x conversion every 1 hour.

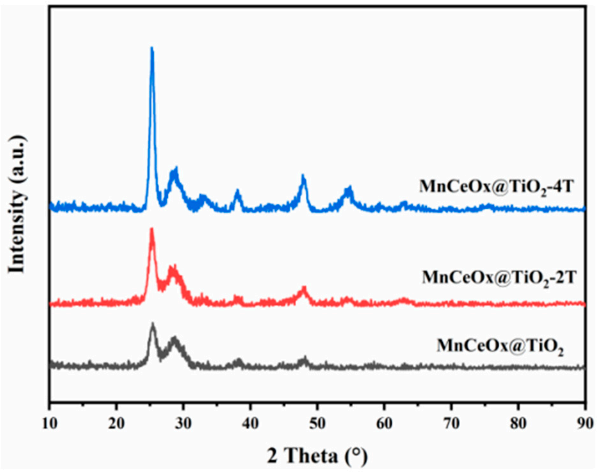

Figure S1. PXRD patterns of $MnCeO_x@TiO_2$ with the addition of different amounts of tourmaline.

Table S1. Comparison of the catalytic performance with previous work.

Catalysts	Preparation methods (calcination temperature)	Reaction conditions	Highest NO_x conversion/N_2 selectivity (temperature range)	Sources
MnO_x	Co-precipitation (100 ˚C)	0.05% NH_3, 0.05% NO, 3%O_2	100% (100–150 ˚C)	S1
MnO_x	Precipitation (350 ˚C)	0.05%NH_3, 0.05% NO, 5%O_2	100%/>82% (100–150 ˚C)	S2, S3
MnO_2	Calcination (400 ˚C)	0.055% NH_3, 0.055% NO, 2%O_2	97%/55% (177 ˚C)	S4
Manganese ore	-----	0.045% NH_3, 0.04% NO, 3%O_2	100% (150–250 ˚C)	S5
MnOx–CuO	Co-precipitation (350 ˚C)	0.05% NH_3, 0.05% NO, 5% O_2	100% (50–200 ˚C)	S6-S8
MnOx–FeOx	Co-precipitation (500 ˚C)	0.1% NH_3, 0.1% NO, 2% O_2	100% (120–180 ˚C)	S9
MnOx–FeOx–TiO_2	Co-precipitation (400 ˚C)	0.05% NH_3, 0.05% NO, 5%O_2	100%/>90% (200–300 ˚C)	S10, S11
MnOx–CeO_2	Co-precipitation (650 ˚C)	0.1% NH_3, 0.1% NO, 2%O_2	100% (120–150 ˚C)	S7, S8
MnOx–SnO_2	Redox co-precipitation (500 ˚C)	0.05% NH_3, 0.05% NO, 3% O_2	100% (120–200 ˚C)	S12
MnOx/Al_2O_3	Pore volume impregnation (500 ˚C)	0.055% NH_3, 0.055% NO, 2% O_2,	95%/>65%(150–250 ˚C)	S13
MnOx/TiO_2	Impregnation (400 ˚C)	0.04% NH_3, 0.04% NO, 2% O_2	95%/72% (170 ˚C)	S14
MnOx/TiO_2	Impregnation (400 ˚C)	0.05% NH_3, 0.05% NO, 3% O_2	100% (150–200 ˚C)	S15
MnOx/TiO_2	Sol–gel (500 ˚C)	0.1% NH_3, 0.1% NO, 3% O_2	100% (120–200 ˚C)	S16
MnOx–CeO_2–Nb_2O_5/cordierite	Co-precipitation (650 ˚C)	0.2% NH_3, 0.1% NO, 10% O_2	80%/96% (200 ˚C)	S17
MnOx/AC/C	Impregnation (400 ˚C)	0.055% NH_3, 0.05% NO, 3% O_2	95% (250 ˚C)	S18
$MnCeO_x$@TiO_2-T2	Co-precipitation (500 ˚C)	0.05% NH_3, 0.05% NO, 5% O_2	100% (110-200 ˚C)	This work

Table S2. Main composition of tourmaline (wt. %)

成分	SiO_2	Al_2O_3	Fe_2O_3	B_2O_3	FeO	H_2O	Na_2O	MgO	TiO_2	Na_2O
电气石	33.54	32.98	16.32	11.21	2.88	2.51	0.71	0.49	0.28	0.01

References:

[S1] X.L. Tang, J.M. Hao, W.G. Xu, J.H. Li, Catal. Commun. 8 (2007) 329.

[S2] M. Kang, E.D. Park, J.M. Kim, J.E. Yie, Appl. Catal. A: Gen. 327 (2007) 261.

[S3] M. Kang, T.H. Yeon, E.D. Park, J.E. Yie, J.M. Kim, Catal. Lett. 106 (2006) 77.

[S4] F. Kapteijn, L. Singoredjo, A. Andreini, J.A. Moulijn, Appl. Catal. B: Environ. 3 (1994) 173.

[S5] T.S. Park, S.K. Jeong, S.H. Hong, S.C. Hong, Ind. Eng. Chem. Res. 40 (2001) 4491.

[S6] M. Kang, E.D. Park, J.M. Kim, J.E. Yie, Catal. Today 111 (2006) 236.

[S7] G.S. Qi, R.T. Yang, R. Chang, Appl. Catal. B: Environ. 51 (2004) 93.

[S8] G. Qi, R. Yang, J. Catal. 217 (2003) 434.

[S9] R.Q. Long, R.T. Yang, R. Chang, Chem. Commun. (2002) 452.

[S10] F. Liu, H. He, Y. Ding, C. Zhang, Appl. Catal. B: Environ. 93 (2009) 194.

[S11] F.D. Liu, H. He, Catal. Today 153 (2010) 70.

[S12] X. Tang, J. Li, L. Wei, J. Hao, Chinese J. Catal. 29 (2008) 531.

[S13] L. Singoredjo, R. Korver, F. Kapteijn, J. Moulijn, Appl. Catal. B: Environ. 1 (1992) 297.

[S14] P.G. Smirniotis, P.M. Sreekanth, D.A. Pena, R.G. Jenkins, Ind. Eng. Chem. Res. 45 (2006) 6436.

[S15] J. Li, J. Chen, R. Ke, C. Luo, J. Hao, Catal. Commun. 8 (2007) 1896.

[S16] Z.B. Wu, R.B. Jin, Y. Liu, H.Q. Wang, Catal. Commun. 9 (2008) 2217.

[S17] M. Casapu, O. Krocher, M. Elsener, Appl. Catal. B: Environ. 88 (2009) 413.

[S18] X. Tang, J. Hao, H. Yi, J. Li, Catal. Today 126 (2007) 406.
